# AI-Enhanced Predictive Modeling for Identifying Depression and Delirium in Cardiovascular Patients Scheduled for Cardiac Surgery

**DOI:** 10.3390/diagnostics14010067

**Published:** 2023-12-27

**Authors:** Karina Nowakowska, Antonis Sakellarios, Jakub Kaźmierski, Dimitrios I. Fotiadis, Vasileios C. Pezoulas

**Affiliations:** 1 Department of Old Age Psychiatry and Psychotic Disorders, Medical University of Lodz, 90-419 Lodz, Poland; karina.nowakowska@umed.lodz.pl (K.N.); jakub.kazmierski@umed.lodz.pl (J.K.); 2 Laboratory of Biomechanics and Biomedical Engineering, Department of Mechanical and Aeronautics Engineering, University of Patras, 26504 Patras, Greece; asakellarios@upatras.gr; 3 Unit of Medical Technology and Intelligent Information Systems, Department of Materials Science and Engineering, University of Ioannina, 45110 Ioannina, Greece; fotiadis@uoi.gr; 4 Biomedical Research Institute—FORTH, University Campus of Ioannina, 45110 Ioannina, Greece

**Keywords:** depression, cardiovascular disease, prediction, explainable artificial intelligence (AI)

## Abstract

Several studies have demonstrated a critical association between cardiovascular disease (CVD) and mental health, revealing that approximately one-third of individuals with CVD also experience depression. This comorbidity significantly increases the risk of cardiac complications and mortality, a risk that persists regardless of traditional factors. Addressing this issue, our study pioneers a straightforward, explainable, and data-driven pipeline for predicting depression in CVD patients. Methods: Our study was conducted at a cardiac surgical intensive care unit. A total of 224 participants who were scheduled for elective coronary artery bypass graft surgery (CABG) were enrolled in the study. Prior to surgery, each patient underwent psychiatric evaluation to identify major depressive disorder (MDD) based on the DSM-5 criteria. An advanced data curation workflow was applied to eliminate outliers and inconsistencies and improve data quality. An explainable AI-empowered pipeline was developed, where sophisticated machine learning techniques, including the AdaBoost, random forest, and XGBoost algorithms, were trained and tested on the curated data based on a stratified cross-validation approach. Results: Our findings identified a significant correlation between the biomarker “sRAGE” and depression (r = 0.32, *p* = 0.038). Among the applied models, the random forest classifier demonstrated superior accuracy in predicting depression, with notable scores in accuracy (0.62), sensitivity (0.71), specificity (0.53), and area under the curve (0.67). Conclusions: This study provides compelling evidence that depression in CVD patients, particularly those with elevated “sRAGE” levels, can be predicted with a 62% accuracy rate. Our AI-driven approach offers a promising way for early identification and intervention, potentially revolutionizing care strategies in this vulnerable population.

## 1. Introduction

Cardiovascular disease (CVD) is the leading cause of death worldwide [1]. In the case of multivessel coronary artery disease (CAD), coronary artery bypass graft (CABG) is one of the options for revascularization. In parallel, depression is the third leading cause of nonfatal health loss globally [2]. It has been reported that depression and CVD present a bidirectional relationship in which a CVD patient is more likely to be depressive and vice versa [3,4]. The more severe the depression condition, the higher the risk of mortality and other cardiovascular events [5].

Regardless of the shared risk factors, such as age, inflammation, and oxidative stress [6], the link between CVD and depression remains unclear. Extensive research has been performed to better understand such mechanism(s), and new advances may explain, at least in part, why depression and CVD are so closely linked. For example, the association between high levels of inflammatory molecules, including interleukin 6 (IL-6) and C-reactive protein (CRP), and the risk of CVD development has been found [7]. These molecules and their inflammatory pathways are also involved in the pathophysiology of depressive disorder [8]. Another candidate molecule that may play a role in both CVD and depression development is the soluble receptor for advanced glycation end products (sRAGE), which is the immune receptor for proinflammatory mediators [9]. Of note, elderly patients with CVD, especially those with concomitant depressive episodes and/or cognitive impairment, are at risk of delirium. Delirium syndrome may develop at any point of hospitalization; however, it is most frequently two to five days after surgery. Postoperative delirium substantially worsens patients’ prognosis and contributes to higher mortality rates [10]. 

Artificial intelligence (AI) refers to computer systems designed to imitate and enhance intelligent human behavior [11]. Intelligent computer programs have been implemented to solve complex problems in almost every field of life, including medicine [12]. AI, with its ability to analyze big data in healthcare, make predictions, and learn from patterns [13], has the potential to revolutionize the field of medicine by improving the quality of care, providing diagnosis at an earlier stage of diseases more accurately, reducing costs, predicting the most appropriate course of action for a patient, and reducing the number of medical errors [14,15]. In recent years, machine-learning-based methodologies have been developed to predict depression due to the increased availability of data. For example, depression was predicted with 86.20% accuracy by employing the random forest (RF) classifier and using data from 6588 patients, including hundreds of items relating to sociodemographic characteristics, health status, status of economic activity, residence, pension, insurance, living expenses, annual family income, whole fortune, debt, living condition, lifestyle, basic living allowance, use of welfare services, subjective satisfaction, family relationship, and mental health [16]. RF also presented the highest accuracy in another study focused on a geriatric population. In that case, the predictive model had 91% accuracy applied to an external validation dataset [17]. In a similar way, many other studies have been presented with the general aim of diagnosis or prediction of depression under different populations or pathological conditions [18,19,20,21].

In this work, we aimed to predict both depression and postoperative delirium among patients who underwent CABG. To this purpose, data from 224 patients were collected, and an expert psychiatrist performed neuropsychiatric assessment before and after the CABG procedure. An AI-empowered pipeline was developed to classify patients at higher risk for depression and delirium, employing and testing three classifiers: AdaBoost, RF, and XGBoost. The novelties of this work are that, for the first time, we predicted the depressive episodes in a particular group of patients who had received CABG treatment and considered confounding factors (cognitive status assessed with the use of the Mini-Mental State Examination (MMSE)) and anemia (hemoglobin concentration < 10 mg/dL for female and <12 mg/dL for male)). Moreover, our results are explainable through the following AI pipeline.

## 2. Materials and Methods

### 2.1. Dataset

A total of 224 adult patients who were qualified for isolated CABG surgery or CABG surgery with cardiac valve repair or replacement (CVR) in the Department of Cardiac Surgery at the Central Clinical Hospital of the Medical University of Lodz, Poland, were eligible for the study. The exclusion criteria were as follows: unstable general condition of the patient, diagnosis of dementia before surgery, delirium diagnosed in the week preceding the procedure, surgery other than CABG or CABG with CVR, chronic inflammatory or autoimmune diseases, use of corticosteroids, cytokine/anticytokine treatment 6 months before surgery, patients on dietary supplements, active alcohol or other substance addiction (abstinence period shorter than 3 months), severely impaired hearing or vision, illiteracy, and death during surgery or in the first five days after surgery. All subjects signed their informed consent for inclusion before participating in the study. The study was conducted in accordance with the Declaration of Helsinki, and the protocol was approved by the Ethics Committee of the Medical University of Lodz, Poland (RNN/95/17/KE 14.03.2017). The study population was examined by a psychiatrist the day prior to the scheduled operation, and a diagnosis of MDD and anxiety disorders was established on the basis of the Diagnostic and Statistical Manual of Mental Disorders (DSM-5) criteria [22]. The MMSE and the clock drawing test (CDT) were performed to evaluate the global cognitive status. The Confusion Assessment Method for the Intensive Care Unit (CAM-ICU) and the Memorial Delirium Assessment Scale (MDAS) (cut-off of 10) were used in parallel to assess the presence of delirium after surgery [23,24,25].

The CAM-ICU is a short test for the diagnosis of delirium. It is characterized by no requirement for verbal communication from the patient, allowing it to be administered in patients undergoing invasive mechanical ventilation and orotracheal intubation [23]. The MDAS is a 10-item, 4-point clinician-rated scale (possible range 0–30) designed to quantify the severity of delirium in medically ill patients. Scale items assess disturbances in arousal and level of consciousness as well as memory, attention, orientation, disturbances in thinking, and psychomotor activity [25]. Patients were assessed by a psychiatrist once a day within the first 5 days after cardiac surgery. Before each examination, the level of sedation/arousal was assessed using the Richmond Agitation Sedation Scale (RASS) [26]. If the RASS was above −4 (−3 through to +4), the assessment with CAM-ICU was administered. However, if the patient scored −4 or −5 on the RAAS during the assessment, which corresponds to deep sedation, the evaluation was stopped and repeated later. In the course of the diagnostic process, nurses and doctors were interviewed and/or clinical notes were interrogated for mention of delirium diagnosis or delirium symptoms. If there was an inconsistency between the diagnostic tools regarding the delirium diagnosis, the final consensus was established within the study team physicians by collecting information from all available sources.

The venous blood samples were taken from the patients twice during the study: the day before the surgery and the first day after the operation between 07:00 and 09:00 a.m. The samples were centrifuged at 7000 rpm for 10 min, and the serum was frozen at −80 °C until biochemical parameters were determined. The levels of sRAGE, MCP-1, and hsCRP were measured in serum with an ELISA kit (BioVendor, Brno, Czech Republic, for sRAGE; R&D, Boston, MA, USA, for MCP-1; and DRG International, Springfield Township, NJ, USA, for hsCRP), and the antioxidant activity was measured with an antioxidant assay kit (Cayman Chemical, Ann Arbor, MI, USA).

The protein concentration of the collected samples was determined using the standard curve and the Stat-Matic Plate Washer II from Sigma-Aldrich, St. Louis, MO, USA. The absorbance was read using the VICTORTM X4 multifunctional microplate reader from Perkin Elmer, Waltham, MA, USA. The immunoenzymatic ELISA test results were analyzed using the WorkOut 2.5 software. The mean concentration of protein per mL was determined by referring to the four-parameter logistic (4-PL) curve. To assess the antioxidant activity, the lag time by antioxidants was measured against the myoglobin-induced oxidation of 2,2′-azino-di(3-ethylbenzthiazoline-6-sulfonic acid) (ABTS) with H_2_O_2_. The assay relies on the ability of antioxidants in the sample to inhibit the oxidation of ABTS to ABTS+ by metmyoglobin. The absorbance at 405 nm can be used to measure the amount of ABTS+ produced. During the reaction, the antioxidants in the sample reduced the absorbance at 405 nm proportionally to their concentration. The capacity of the sample’s antioxidants to prevent ABT oxidation was compared to that of Trolox, a water-soluble tocopherol analog, and was measured in millimolar Trolox equivalents.

The laboratory determinations were conducted by laboratory diagnosticians who were blinded to clinical data.

### 2.2. Proposed Workflow of Depression and Delirium Prediction

**Figure 1:**
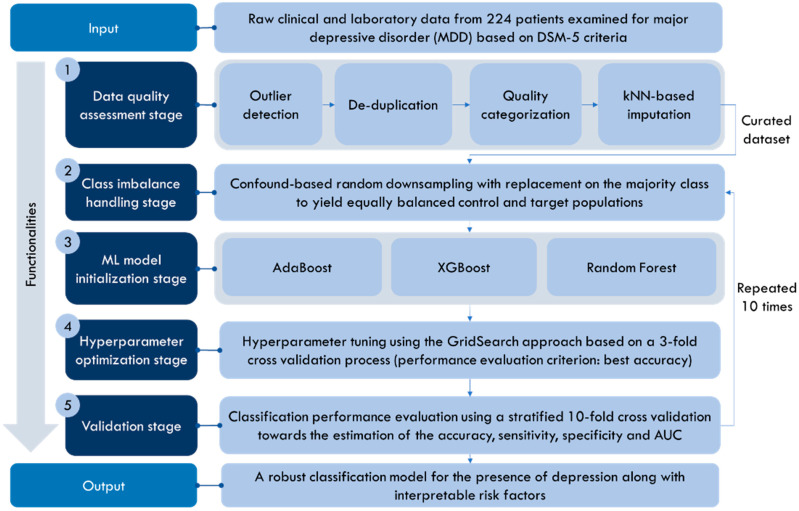
The proposed workflow of depression and delirium prediction.

The proposed workflow of depression and delirium prediction is depicted in Figure 1. It consists of five stages, namely, (i) the data quality assessment stage, (ii) the class imbalance handling stage, (iii) the ML model initialization stage, (iv) the hyperparameter optimization stage, and (v) the validation stage. The raw input dataset was the clinical and laboratory dataset from the Central Clinical Hospital of the Medical University of Lodz in Poland, which was described in the previous section. In the data quality assessment stage, any outliers and duplicated fields were automatically removed from the data. The quality of the features was categorized into three states, and kNN-based imputation was applied where applicable. Confound-based random downsampling with replacement was applied to the majority class (patients without depression) to produce a subset of equally sized control and target populations where the confound factors remained stable. Three different boosting and bagging machine learning algorithms (AdaBoost, XGBoost, and random forest) were then initialized and trained on the extracted subset upon the estimation of the optimal hyperparameters through a GridSearch cross-validation approach. The optimal hyperparameters (i.e., those yielding the highest classification accuracy towards the classification of depression across the 3 folds) were fed into the models, and a stratified 10-fold cross-validation process was finally applied to estimate the accuracy, sensitivity, specificity, and area under the ROC curve (AUC). Stages 2–5 were applied 10 times to reduce biases. The output of the proposed workflow was a robust classification model for the presence of depression along with interpretable risk factors.

#### 2.2.1. Data Quality Assessment (Data Curation) Stage

An advanced data curation pipeline presented in a previous study [27] was applied to automatically remove outliers and duplicated fields and impute missing values where necessary. The Spearman rank-order correlation coefficient was calculated for each pair of feature values, and the Levenshtein distance was computed for each pair of feature labels to identify features with the same lexical and contextual similarity. The local outlier factor (LOF) [28] was used to identify areas with increased density across the feature distribution, implying potential outliers. The isolation forest (IF) algorithm [29] was also trained on the nonmissing data without any outliers to identify contaminated features. The quality of the features was classified as “good” (no missing values), “fair” (less than 30% missing values), and “bad” (more than 30% missing values). The kNN imputer [30] was applied only for features with a “fair” quality state.

#### 2.2.2. Class Imbalance Handling Stage

Random downsampling with replacement was applied to the majority population to yield an equally balanced control and target population for the training process. The outcome was set to depression (with “0” denoting the absence of depression and “1” indicating the presence of depression). The downsampling process was repeated 10 times. In each round, the randomly sampled controls were matched with the target population according to the MMSE (Mini-Mental State Examination) and anemia (hemoglobin concentration < 10 mg/dL) using either the nonparametric Wilcoxon rank-sum test in the case of continuous features (a Shapiro–Wilk test was first applied to evaluate the normality of the data; in the case of a normal distribution, the Student’s *t*-test was applied instead) or the chi-square/Fisher’s exact test in the case of discrete features.

#### 2.2.3. ML Model Initialization Stage

Boosting and bagging supervised machine learning algorithms, including AdaBoost (adaptive boosting) [31], XGBoost (extreme gradient boosting) [32], and random forest (RF) [33], were deployed for the development of a binary classification model for the presence of depression.

The gradient boosting algorithm [32] combines a set of weak learners into a stronger classifier, where on each boosting round, the algorithm minimizes the gradient of a loss function to optimize the overall performance of the classifier. At step i, the gradient boosting classifier seeks a weak learner, say $f_{i}\left(d\right)$, so that

(1) $$F_{i}\left(d\right)=F_{i-1}\left(d\right)+f_{i}\left(d\right).$$

Assuming that $\tilde{y}$ is the predicted value at step i, the goal is to minimize the cost function:

(2) $$F_{i}\left(d\right)=F_{i-1}\left(d\right)+argmin_{f}\left(\overset{n}{\underset{j=1}{\sum }}L\left({\tilde{y}}_{j},F_{i-1}\left(d_{j}\right)+f_{i}\left(d_{j}\right)\right)+r\right),$$

where ${\tilde{y}}_{j}$ is the predicted value for the input sample $d_{j}$, $L\left(.\right)$ is the error loss function, n is the number of samples, and r is a regularization term that is used to avoid overfitting. In the case of tree learners, the regularization term is defined as follows:

(3) $$r=\gamma M+\frac{1}{2}\lambda \overset{J}{\underset{j=1}{\sum }}w_{j}{}^{2},$$

where γ  and λ are scalars, M is the number of leaves in each tree learner, and w is the weight on the leaves. The implementation was performed in Python 3.6.3 using XGBoost.

The AdaBoost (adaptive boosting) classifier combines a set of N-weak learners in a sequential error reduction fashion, where the final output of the classifier is a weighted sum of the weak classifiers. The final classifier can be expressed as follows:

(4) $$F_{N}\left(d\right)=\overset{N}{\underset{i=1}{\sum }}f_{i}\left(d\right),$$

where d is the input vector, $F_{N}\left(d\right)$ is the final classifier, and N is the number of boosting rounds.

The random forest (RF) algorithm adopts a bagging strategy according to which a voting approach is used to combine the decisions across a set of individual decision trees, which are trained on randomly selected subsets of the original dataset to reduce further biases introduced by the conventional decision tree models and provide more accurate classification outcomes.

#### 2.2.4. Hyperparameter Optimization Stage

The following set of hyperparameters were evaluated for each boosting classifier under a 3-fold cross-validation process using the GridSearch approach: (i) CatBoost: number of trees (50–200), learning rate (0.001–0.3), and tree depth (3–10); (ii) XGBoost: learning rate (0.01–0.3), maximum depth (3–10), and subsample ratio (0.7–1); and (iii) AdaBoost: number of base estimators (50–200), algorithm (SAMME or SAMME.R), and learning rate (0.01–0.3).

#### 2.2.5. Validation Stage

The best hyperparameters from the previous section were introduced into the boosting classifiers. A stratified 10-fold cross-validation was then applied in each successful downsampling iteration to evaluate the classification performance of the boosting schemas by estimating the accuracy, sensitivity, area under the ROC curve (AUC), and specificity scores.

## 3. Results

The demographic characteristics of the study group are presented in Table 1. Statistical analysis was initially performed to identify possible correlations between serum biomarkers and depression among CABG individuals. Table 2 presents the correlations between biomarkers.

The random forest classifier and gradient boosted tree (GBT) presented the highest performance in relation to depression diagnosis and delirium prediction, respectively (Table 3).

**Figure 2:**
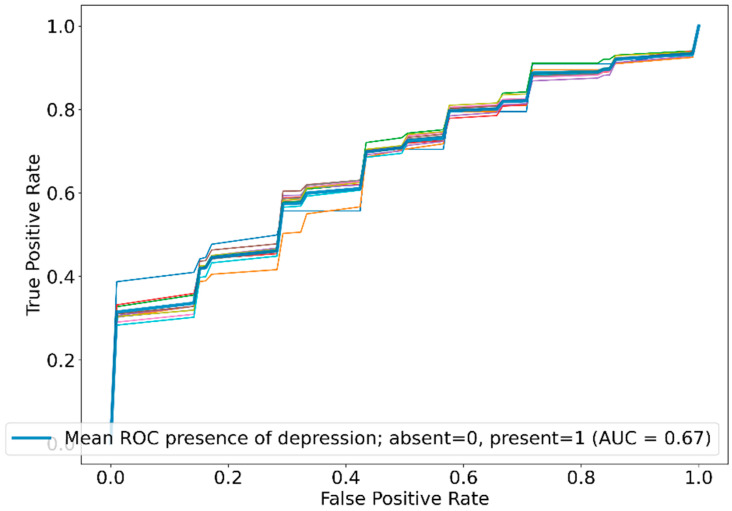
Mean ROC curve for the diagnosis of depression with the use of the random forest algorithm averaged across multiple runs.

The ROC curve analysis of the iterative runs using RF is presented in Figure 2, where the variations among different runs were insignificant.

**Figure 3:**
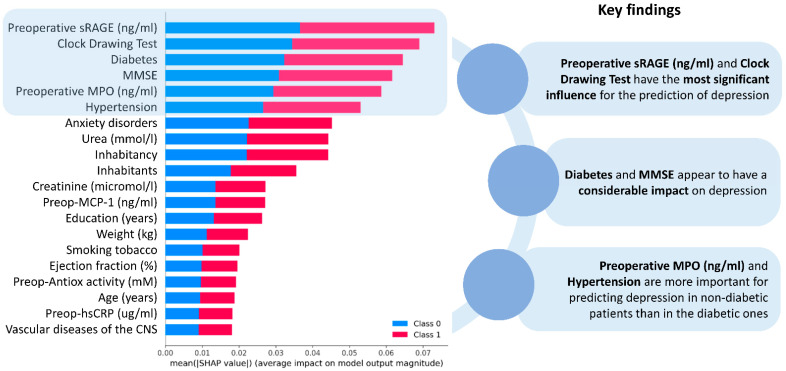
Feature importance scale in depression detection. MPO: myeloperoxidase, sRAGE: soluble receptor for advanced glycation end products, MMSE: Mini-Mental State Examination (points), MCP-1: monocyte chemoattractant protein-1, hsCRP: high-sensitivity C-reactive protein, CNS: central nervous system.

**Figure 4:**
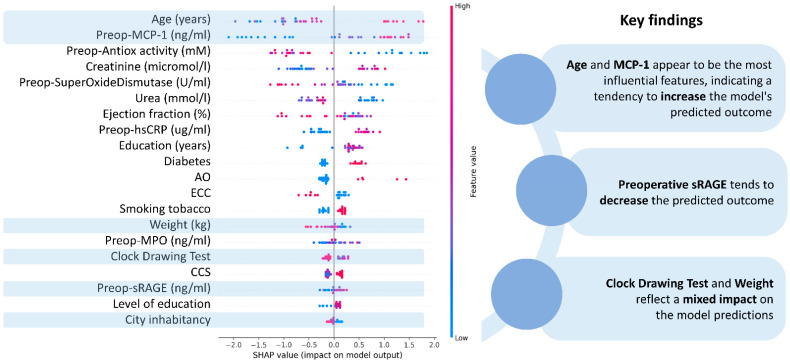
Feature importance values in delirium prediction. CCS: Canadian Cardiovascular Society class grading of angina pectoris, sRAGE: soluble receptor for advanced glycation end products, MPO: myeloperoxidase, MCP-1: monocyte chemoattractant protein-1, hsCRP: high-sensitivity C-reactive protein, ECC: extracorporeal circulation, AO: Peripheral artery.

Feature importance has been calculated for the detection of depression among patients scheduled for CABG (Figure 3) and postoperative delirium prediction (Figure 4). It has been found that preoperative sRAGE is the most significant feature in depression prediction. Other factors that contribute to the outcome were cognitive status (CDT score) and diabetes. Advanced age and increased preoperative MCP-1 levels are the most important in delirium prognosis.

## 4. Discussion

In this work, for the first time, we present an AI-empowered pipeline to detect patients with a diagnosis of depression and delirium in a group of individuals scheduled for CABG. For this purpose, 224 patients were included in the current study. Blood samples were collected for sRAGE, MCP, hsCRP, antioxidant capacity, and SOD, and psychiatric assessment was performed before and after CABG. Employing the RF classifier, CABG patients with depression could be detected with 62% accuracy (AUC = 0.67) on the basis of the preoperative sRAGE levels, whereas the use of GBT predicted delirium with 72% accuracy.

Advanced glycation end products (AGEs) are heterogeneous groups of irreversible adducts formed from the nonenzymatic glycation and glycoxidation of proteins and nucleic acid with reducing sugars [34]. The interaction between AGEs and their cell-bound receptor for advanced glycation end products (RAGE) plays a vital role in oxidative stress and the innate immune response. RAGE may stimulate proinflammatory processes that contribute to atherosclerosis by reducing nitric oxide (NO) levels. NO protects blood vessels through vasodilatation, decreased platelet aggregation and activation, and increased production of reactive oxygen species (ROS) [35,36,37,38]. ROS activates nuclear factor kappa-B (NF-kB), which then activates numerous proinflammatory genes of cytokines, such as tumor necrosis factor-α (TNF-α) and interleukin (IL)-1, IL-2, IL-6, IL-8, and IL-9 [39,40]. The leading cause of CVD is atherosclerosis, in which plaques are raised by molecular changes induced by cytokines, hormones, growth factors, and oxidative species, mainly due to the interaction between endothelial cells, LDLs, and macrophages [41].

Studies conducted so far indicate the relationship between oxidative stress and the pathogenesis of depression and CVD [42,43,44]. A meta-analysis published in 2015 found that oxidative stress, measured by 8-hydroxy-2′-deoxyguanosine (8-OHdG) and F2-isoprostanes, increased in depression [44].

Oxidative stress is defined as an early causative factor of CVD. The influence of ROS on underlying endothelial molecules, which can promote apoptosis, necrosis, and therefore thrombosis of atherosclerotic plaques, makes oxidative stress a crucial hallmark of CVD [45].

It has been observed that both depression and postoperative delirium are common among CAD (coronary artery disease) patients, which may indicate a correlation between the two. In addition, our previous study on the development of delirium revealed that patients with major depressive disorder (MDD) and higher levels of cortisol before surgery are more vulnerable to postoperative delirium [46]. However, it should be noted that only about half of the patients with depression have increased cortisol levels [47].

Therefore, we decided to investigate other potential mechanisms that may explain the link between MDD and delirium.

The role of the soluble receptor for advanced glycation end products is still unclear. sRAGE is found to be negatively associated with inflammation by binding RAGE ligands and thus blocking their interaction with membrane-bound RAGE [48]. Exogenous administration of soluble RAGE has been shown to reduce oxidative stress markers in animal models of vascular dysfunction [49,50]. Low levels of plasma sRAGE in nondiabetic patients with coronary artery disease compared to control subjects have been reported [51]. However, elevated sRAGE levels were observed in patients with type 1 and type 2 diabetes and patients with renal failure and were associated with ischemic incidents in CVD individuals [52,53]. Despite the high level of sRAGE, which is supposed to be protective through its anti-inflammatory effects, atherosclerosis develops in diabetic patients [54].

A rise in sRAGE levels has also been linked to a higher mortality risk in hemodialysis and peritoneal dialysis patients [55]. Significantly lower levels of sRAGE have been described in patients with hypercholesterolemia, hypertension, chronic obstructive pulmonary disease, Alzheimer’s disease, and vascular dementia [56,57,58,59].

In the current study conducted in a population scheduled for CABG, which means with advanced cardiovascular dysfunction, the sRAGE expression before surgery was higher in the group of participants with depression than those without a diagnosis of MDD. This may reflect the protective mechanisms of sRAGE, according to which overexpression of sRAGE regulates inflammation and reduces cell damage related to oxidative stress among patients with CVD and concomitant diagnosis of depression. The differences in sRAGE levels depending on the medical condition diagnosed may result from different pathogenesis of specific disorders. For instance, in conditions with RAGE-mediated inflammation and oxidation, higher sRAGE concentration may constitute a protective factor, whereas in diseases with proinflammatory mechanisms and pathophysiology unrelated to RAGE or in the case of ineffective protective processes, the sRAGE levels are lower.

Our previous study dedicated to the pathogenesis of delirium revealed that patients with MDD and increased levels of cortisol prior to surgery were more likely to develop postoperative delirium [46]. Elevated cortisol values have been reported in both depressed and CVD patients as a consequence of increased hypothalamic–pituitary–adrenal (HPA) axis reactivity [47,60]. These correlations suggest that oxidative stress and inflammation promote depression and CVD, giving a feasible common link between them.

Depression is associated with complications for optimal CVD management, including low adherence to a healthy lifestyle and to taking medications in accordance with medical recommendations [61]. MDD increases mortality, disability, and healthcare expenditure and reduces quality of life among patients with CVD [62,63]. Unfortunately, strategies for screening and treating depression are poorly implemented in patients with CVD. The inclusion of sRAGE testing in standardized screening pathways for depression in CVD patients would offer the possibility of early and more precise identification and optimal treatment of depression to improve health outcomes.

The present analysis also revealed the variables with the highest significance in delirium prognosis. Advanced age, higher MCP-1 concentration, and lower antioxidant activity had the highest accuracy in predicting the development of postoperative delirium. More advanced age is consistently reported as an unmodifiable risk factor for delirium [64]. In addition, both increased MCP-1 level and decreased antioxidant capacity were associated with delirium development in our previous study using regression modeling as the main statistical method [65].

Only selected confounders were included in the statistical analysis, which can be assumed to be a potential limitation of the present study. However, the novel statistical approach used in the current analysis provided valid results, even with the limited number of potential confounders considered. Furthermore, the sRAGE levels were evaluated only once, not at different time points, and were not compared to individuals with depression resolution. This issue could be the aim of further studies in the field.

## 5. Conclusions

This study marks a significant advancement in the field of cardiology and mental health, presenting, for the first time, an AI-empowered pipeline for detecting depression in patients undergoing coronary artery bypass graft (CABG) surgery. By analyzing a cohort of 224 patients, we identified key biomarkers, including sRAGE, MCP, hsCRP, antioxidant capacity, and SOD, that can be coupled with psychiatric assessments to assess the risk of depression and delirium pre- and post-CABG. Our findings highlight the potential of sRAGE as a biomarker for depression in this patient group, with the random forest classifier achieving a 62% accuracy rate (AUC = 0.67) in predicting depression based on preoperative sRAGE levels. Concurrently, the gradient boosted tree (GBT) model effectively predicted delirium with 72% accuracy. The study underlines the intricate relationship between oxidative stress, inflammation, and the pathogenesis of both depression and cardiovascular disease (CVD). sRAGE emerges as an indicator of protective anti-inflammatory mechanisms in some contexts while being associated with increased disease severity and mortality risk in others. This duality underscores the complexity of CVD and depression pathogenesis and the necessity of nuanced approaches in their management. The implications of this research are profound, suggesting that incorporating sRAGE testing into standardized depression screening for CVD patients could significantly enhance the early detection and treatment of depression, potentially improving patient outcomes. Furthermore, the study sheds light on factors influencing postoperative delirium, with advanced age, MCP-1 concentration, and antioxidant activity being critical predictors. In the future, it would be useful to determine the level of sRAGE at different time points to examine its dynamics. This would help establish whether there is a relationship between sRAGE levels and the severity of depressive symptoms. Additionally, it would be interesting to observe whether sRAGE levels return to normal after achieving remission in depression and how long this takes. Establishing such a relationship would help improve the monitoring of patients with MDD and CVD, a vulnerable group requiring special attention. These patients often have comorbidities and are on multiple medications, increasing the risk of drug interactions. The use of AI to diagnose and monitor patients can significantly enhance and facilitate medical care, reducing the number of medical errors.

While the study presents groundbreaking findings, it also acknowledges limitations, including the singular evaluation of sRAGE levels and the exclusion of certain confounders. Future research could expand upon these findings, exploring the dynamics of sRAGE levels over time and in patients experiencing depression resolution. In conclusion, this study offers a promising step towards a more integrated and precise approach to diagnosing and managing depression in patients with CVD, potentially paving the way for improved clinical outcomes and patient care.

## Figures and Tables

**Table 1:** Demographics and clinical characteristics of the population.

Characteristic	Mean or *N*	SD or %
Age (years)	66.66	7.06
Gender (Male)	171	80%
Education (years)	11.53	3.37
Weight (kg)	81.69	12.38
Height (cm)	170.22	8.20
Presence of anxiety disorders	14	7%
Alcohol addiction	17	8%
Diabetes	74	34%
Hypertension	176	82%
Peripheral arterial disease	31	14%
Vascular diseases of the CNS	24	11%
Other diseases of the CNS (epilepsy or head injuries)	8	4%
Asthma	7	3%
Chronic obstructive pulmonary disease	14	7%
Smoking tobacco	94	44%
Anemia (Hb 10 mg/dL for female; 12 mg/dL for male)	32	15%
Creatinine > 1.2 mg/dL	31	14%
Atrial fibrillation	28	13%
Pacemaker	4	2%
Significant ventricular arrhythmias	2	1%
Ejection fraction %	51.61	10%
CCS score	2.39	0.74
NYHA grade	2.17	0.70
Clock drawing test (points)	5.79	2.40
Mini-Mental State Examination (points)	27.60	2.10
Antioxidant activity (mM) before surgery	2.30	1.12
Superoxide dismutase (U/mL) before surgery	3.00	1.58
sRAGE (ng/mL) before surgery	1.02	0.74
MPO (ng/mL) before surgery	321.54	238.98
MCP-1 (ng/mL) before surgery	423.17	184.46
hsCRP (ug/mL) before surgery	10.67	23.14
Urea (mmol/L) before surgery	6.81	2.07
Creatinine (mcmol/L) before surgery	88.55	23.80
Intraoperative circulatory support	57	27%
Intraoperative resuscitation	3	1%
Intraoperative steroid use	2	1%
Postoperatively (pCO_2_ ≥45 mmHg)	50	23%
Postoperatively (pO_2_ ≤ 60 mmHg)	31	14%
Postoperative hyperthermia	23	11%
Massive postoperative transfusion (>4 units)	11	5%
Plasma transfusion (≥1 unit)	35	16%
Reoperation	13	6%
Urgent postoperative angioplasty	1	0%
Length of stay in the ICU (days)	3.71	2.24
Hospitalization time (days)	12.83	6.98
ECC; in case of no (surgery OPCAB)	165	77%
The highest MDAS score	7.72	4.59
Delirium diagnosis	61	34%
Day after surgery at which delirium was diagnosed	28	13%
Presence of depression	34	16%

CNS: central nervous system, CCS: Canadian Cardiovascular Society class grading of angina pectoris, NYHA: New York Heart Association, sRAGE: soluble receptor for advanced glycation end products, MPO: myeloperoxidase, MCP-1: monocyte chemoattractant protein-1, hsCRP: high-sensitivity C-reactive protein, pCO_2_: partial pressure of carbon dioxide, pO_2_: partial pressure of oxygen, ICU: intensive care unit, ECC: extracorporeal circulation, OPCAB: off-pump coronary artery bypass, MDAS: Memorial Delirium Assessment Scale, Hb: hemoglobin concentration.

**Table 2:** Correlation between preoperative biomarkers and depression.

Biomarker	Correlation Coefficient	*p*-Value
MPO (ng/mL)	0.14	0.29
Antioxidant activity (mM)	−0.11	0.40
Superoxide dismutase (U/mL)	−0.11	0.25
sRAGE (ng/mL)	0.32	0.04
MCP-1 (ng/mL)	0.06	0.62
hsCRP (ug/mL)	0.08	0.54

MPO: myeloperoxidase, sRAGE: soluble receptor for advanced glycation end products, MCP-1: monocyte chemoattractant protein-1, hsCRP: high-sensitivity C-reactive protein.

**Table 3:** Detection of depression and delirium with the use of different classifiers.

	Classifier	Accuracy	Sensitivity	Specificity	AUC
Depression	**RF**	**0.614**	**0.701**	**0.528**	**0.671**
	GBT	0.565	0.598	0.532	0.571
	XGBoost	0.558	0.610	0.505	0.582
	AdaBoost	0.542	0.715	0.367	0.599
Delirium	**GBT**	**0.722**	**0.719**	**0.723**	**0.781**
	RF	0.709	0.757	0.657	0.787
	XGBoost	0.669	0.670	0.664	0.744
	AdaBoost	0.583	0.920	0.245	0.727

## Data Availability

Data used in this manuscript could be available upon contact the PIs of the clinical study.
